# Non-linear convective flow of the thin film nanofluid over an inclined stretching surface

**DOI:** 10.1038/s41598-021-97576-x

**Published:** 2021-09-15

**Authors:** Anwar Saeed, Poom Kumam, Saleem Nasir, Taza Gul, Wiyada Kumam

**Affiliations:** 1 Faculty of Science, Center of Excellence in Theoretical and Computational Science (TaCS-CoE), King Mongkut’s University of Technology Thonburi (KMUTT), 126 Pracha Uthit Rd., Bang Mod, Thung Khru, Bangkok, 10140 Thailand; 2 Department of Medical Research, China Medical University Hospital, China Medical University, Taichung, 40402 Taiwan; 3 Department of Mathematics, City University of Science and Information Technology, Peshawar, Pakistan; 4 Applied Mathematics for Science and Engineering Research Unit (AMSERU), Program in Applied Statistics, Department of Mathematics and Computer Science, Faculty of Science and Technology, Rajamangala University of Technology Thanyaburi, Thanyaburi, Pathumthani 12110 Thailand

**Keywords:** Engineering, Mathematics and computing

## Abstract

To enhance the surface properties of solids the mechanism of thin films is frequently used. Penetration, degradation, stiffness, illumination, diffusion, absorption, and electric performance are all characteristics of a bulk substance medium that a thin film can improve. In nanotechnology, thin film processing can be extremely useful. Therefore, the time-dependent nonlinearly convective stream of thin film nanoliquid over an inclined stretchable sheet with magnetic effect is investigated in current work. The features of mass and heat transport processes are explained using important factors like thermophoresis and Brownian movement. Nonlinear partial differential equations are obtained to model the time-dependent liquid film flow over an inclined surface, which are then turned into couple ordinary differential equations utilizing appropriate alterations. The results of the computation of the model problem are collected using an analytical approach Homotopy Analysis Method and presented the final finding numerically and graphically. During the flow assessment, the impact of individual flow factors such as magnetic, Brownian, and thermophoresis parameters on regular profiles (temperature, velocity, and concentration) are analyzed and found to be quite remarkable. Furthermore, the consequence of M and N_t_ factors on the velocity, concentration and thermal distribution leads to diminishing conduct. On the other hand, the thermal profile of the liquid film rises in response to the thermophoresis factor. The % wise variation in the skin friction, Nusselt number and Sherwood number versus physical parameters has been obtained and discussed.

## Introduction

Because of its broad applications, the study of mass and heat transport due to the extending surfaces of the liquid film has received a lot of attention lately. Many non-linear problems in fluid mechanics can be studied numerically in depth. A fundamental and fascinating non-linear problem emerged in the case of liquid film stream of both Newtonian as well as non-Newtonian liquids. The perception and structure of various heat transfer and industrial processes equipment are dependent on liquid film motion and heat transport inside the liquid flow. The liquid film layer has a wide range of uses, including an antireflection coating on glasses, diffusion barriers, and liquid sensor technology. Such kinds of liquid film are utilized within capacitors and resistors in the field of the electrical industry. It's also utilized in fiber and wire sheets, amalgam and plastic suspension, food waste eradication, rubber sheets, exchangers, and several other cooling and fluidization applications. The coating systems need a smooth coated surface, lightweight, clarity, and strength to satisfy all these applications' specifications. Besides these applications, Wang^1^ pioneered theoretical studies in liquid film flow by analyzing the characteristics of thin liquid film flow past a non—permeable stretching surface. Whenever the magnitude of the unsteady factor (S) dropped beyond the set of possibilities of S > 2, the similarities approaches were observed to be null. By analyzing asymmetric flow, Usha and Sridharan^2^ reevaluated the work of^1^ and clarified that similarities approaches become inaccessible when the unsteady factor value lies beyond the limits of S > 4. Utilizing the famous model Buongiorno's, Qasim et al.^3^ premeditated heat and mass exchange movement of a nanoliquid film through an unsteady stretched medium. Andersson et al.^4^ tackled the issue of liquid film motion against an unsteadiness stretchable surface in a standard non-Newtonian liquid. They concluded that the power-law index has a greater effect at higher estimations of the unsteadiness factor. The scientists then recognized that in a thin film flow, heat transfer is just as significant as fluid flow dynamics, therefore overcoming the difficulties in the construction of different steam turbines and chemical production machines. Vajravelu et al.^5^ described an unsteady liquid film of Ostwald–de Waele liquid with heat transfer passing through a stretchable surface. Khan^6^ discusses the role of a liquid film in the company of non-Newtonian liquid inertial mechanics. Siddiqui et al.^7^ calculated the HPM (Homotopy Perturbation Method) result of non-Newtonian thin-film liquid flow upon a conveyer belt. The heat transportation of a thin film second-grade liquid in a permeable material via an expanding medium was considered by Noor et al.^8^. Li et al.^9^ explored a continuous thin layer of unsteady MHD liquid stream and transfer of nanofluid heat in the company of heat production (Generation) and thermophoresis factors. Martin et al.^10^ presented a model of film stream for studying gravitational, thin wavy liquid films. Recently several other studies have investigated liquid film motion of Newtonian as well as non-Newtonian liquids experimentally and numerically, for example, Iqbal et al.^11^, Khan et al.^12^, Yusuf et al.^13^ and Tahir et al.^14^.

The study of nanofluids is one of the most important fields within scientific literature because of their incredible ability to improve heat exchange. The word "nanofluid" refers to a colloidal solution composed up of many smaller microparticles of just under 100 nm range. Choi^15^ came up with the term "nanofluid" to produce an innovative form of liquid. Alshomrani et al.^16^ investigated the movement of the MHD nanocomposites across an expanding layer in a permeable material under convective boundary conditions. Sandeep et al.^17^ addressed strengthened thermal performance incorporated with inorganic nanomaterials in liquid film stream of non-Newtonian nanoliquids. The thermal efficiency of nanocomposites is supposed to be determined by influences including the base fluid's heat capacity and thermal ability, the flowrate, the nanofluid's solubility, the amount of colloidal matter as well as their proportions, and the flow structure. In light of the above findings, several researchers and scientist have turned their interest to the study of heat transfer in this recent field for example Khan and Azam^18^, Das et al.^19^, Safwa et al.^20^, Gowda et al.^21^, Khan et al.^22^, Yusuf et al.^23^ and Alsagri et al.^24^. Even so, as nanofluids are nanoparticle suspensions in their base fluids, a robust nanofluid distribution will be helpful in pharmaceutical and biological utilization.

In the field of research and engineering, the importance of MHD (Magneto-hydrodynamics) to the fluid flows problems has been treated seriously by the researchers that have many uses such as energy generator, electromagnetic medicine therapy, turbines, nuclear reactor ventilation and energy exchanges. Anwar et al.^25^ also studied the effects of cross-diffusion on an extended surface during the formation of electromagnetic assets. Khan et al.^26^ suggested the flow of nanofluid with flux radiation and magnetic effects past a wide stretching surface. The characteristics of joule heating, magnet influence and dissipation of the micropolar fluid through an extended surface are considered by Waqas et al.^27^. The effect of MHD on the Casson-Williamson fluid and thermal energy across an extended interface was analyzed by Raju et al.^28^. The thermal transport of Magnetohydrodynamic Casson nanofluid with fleshy media was investigated by Sulochana et al.^29^. Also, various scientists and researchers such as Tlili et al.^30^, Raddy et al.^31^, and Ramzan et al.^32^ published new work on MHD in different configurations and contexts.

Brownian motion effects along with thermophoresis have system components in the scientific and technological field such as fiber optics manufacturing, plastic emulsion, glass cutting, nanoelectronics freezing, catalytic reactors, wire drawing and increased oil extraction. Sheikholeslami et al.^33^ investigated the effect of thermo-phoresis and Brownian moment on the Magnetohydrodynamic nanofluid identified by using FEM. Chamkha and Issa^34^ studied the magnetohydrodynamic movement over the horizontal plane with eccentricities in thermophoresis. Nadeem et al.^35^ examined the Brownian dynamics on magneto-hydrodynamics Maxwell fluid coated by nanomaterials. The nanofluid flow through a stretched medium was investigated in presence of Brownian motion along with thermophoresis by Abdelmalek et al.^36^ and Adeosun et al.^37^. They observed that these outcomes were beneficial for temperatures. In presence of Thermo-phoresis and Brownian motion, Goudarzi et al.^38^ examined the hybrid nanoliquid flow.

The above study analysis reveals that different scholars and scientists analyze the thin film flow of nanoliquid through multiple situations. Nevertheless, nobody in the literature addressed the time-dependent thin nano-fluid film flow through inclined stretching plates with magnetic effect and non-linear convection. Thus, our goal is, therefore, to analyze the incompressible, time dependent 2D thin film nanofluid flow with magnetic influence and non-linear convection. The Brownian motion along with thermophoresis is also taken into account. The main focus has been given to the nonlinear mixed convection and this is the main contribution to the existing literature. The nonlinear systems of equations are solved by employing the HAM method, and the outcomes are described in the form of numerous plots and numerically constructed tabulated tables against different special factors. The following is the outline for this consultation paper. In the second section, the mathematical formula is shown. In section three, the HAM procedure is listed. The results and discussion are covered in section four. Conclusions are found in section five.

## Formulation

**Figure 1:**
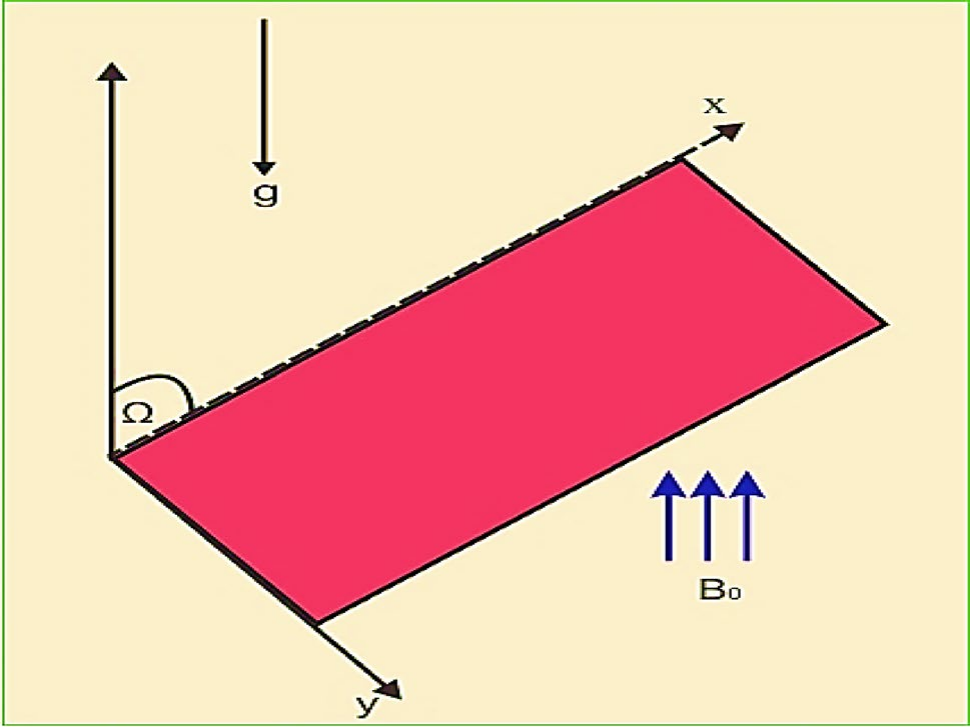
Physical sketch of model problem.

In current theoretical flow model, a 2D time dependent, incompressible, non-linear thin nanofluid film flow of uniform thickness over an inclined stretching surface is consider. Geometry of the modeled problem is exhibited in Fig. 1. We also made certain assumptions in order to organize our study, such as.

1. The stretching sheet at y = 0 move with velocity $U_{w} = \frac{b\,x}{{1 - \gamma t}},$(take γ and b positive constants).
2. The surface temperature distribution $T_{w} (x,t) = T_{0} - T_{r} \left( {\frac{{bx^{2} }}{2v}} \right)(1 - \gamma t)^{{\frac{ - 3}{2}}}$ of the sheet vary with distance x from the slit. Reference temprature *Tr* is (0 < *Tr* < *T*_*0*_).
3. While the concentration field $C_{w} (x,t) = C_{0} - C_{r} \left( {\frac{{bx^{2} }}{2v}} \right)(1 - \gamma t)^{{\frac{ - 3}{2}}}$ of the sheet vary with distance x. Reference concentration *Cr* is (0 < *Cr* < *C*_*0*_).
4. The effect of magnetic field is taken in the form $B(t) = B_{0} \left( {1 - \gamma t} \right)^{{\frac{ - 1}{2}}}$.
5. The characteristics of heat and mass transfer processes are explained with the help of Brownian motion and thermophoresis parameters.

With the help of above norms, the prescribing equations are take the form^3,37^

1 $$ \frac{\partial \,u}{{\partial \,x}} + \frac{\partial \,v}{{\partial \,y}} = \,\,0, $$

2 $$ \begin{aligned} & \rho \left[ {\frac{\partial \,u}{{\partial \,t}} + u\,\,\frac{\partial \,u}{{\partial \,x}} + v\,\,\frac{\partial \,u}{{\partial \,y}}} \right] = \frac{{\partial^{2} u}}{{\partial y^{2} }} + \frac{{\partial^{2} u}}{{\partial x^{2} }} - \sigma B^{2} (t)u \\ & \quad + \rho g{\text{Cos}} \theta \left[ {(T - T_{h} )\left( {\beta_{T} } \right) + (T - T_{h} )^{2} \left( {\beta_{T} } \right)^{2} + (C - C_{h} )\left( {\beta_{C} } \right) + (C - C_{h} )^{2} \left( {\beta_{C} } \right)^{2} } \right], \\ \end{aligned} $$

3 $$ \left( {\rho c_{p} } \right)\left( {\frac{\partial T}{{\partial t}}\,\, + \,\,u\,\,\frac{\partial T}{{\partial x}}\,\, + \,\,v\,\,\frac{\partial T}{{\partial y}}} \right) = K\left[ {\frac{{\partial^{2} T}}{{\partial^{2} y}} + \frac{{\partial^{2} T}}{{\partial^{2} x}}} \right] + \tau \,\,\left[ {\left( {\frac{\partial C}{{\partial y}}.\frac{\partial T}{{\partial y}}} \right)D_{B} + \frac{{D_{T} }}{{T_{h} }}.\left( {\frac{\partial T}{{\partial y}}} \right)^{2} } \right], $$

4 $$ \left[ {\frac{\partial C}{{\partial t}} + u\frac{\partial C}{{\partial x}} + v\frac{\partial C}{{\partial y}}} \right] = D_{B} \left( {\frac{{\partial^{2} C}}{{\partial y^{2} }}} \right) + \left( {\frac{{D_{T} }}{{T_{h} }}} \right)\,\,\frac{{\partial^{2} T}}{{\partial y^{2} }}. $$

### Model physical conditions

The equivalent physical conditions for model problem are:

5 $$ \begin{aligned} & {\text{At}}\,\,y = 0,\,\,\,\,\,\,\,\,\,\,\,\,\,\,\,u = U_{w} ,\,\,\,\,\,\,v = 0,\,T = T_{w} ,\,C = C_{w} , \\ & {\text{At}}\,\,\,\,\,y = h(t),\,\,\,\,\,\,\,\,\frac{\partial u}{{\partial y}} = 0 = \,\,\,\,\frac{\partial T}{{\partial y}}\, = \frac{\partial C}{{\partial y}}\,\,\,\,\,{\text{and}}\,\,\,\,\,v = u\,\,\left( {\frac{\partial h}{{\partial t}}\,} \right). \\ \end{aligned} $$

where t is time, $u$ is velocity in $x$-direction, $v$ is velocity in $y$-direction, h is thickness of thin film, $\beta_{T}$ is the thermal expansion coefficient, $\beta_{C}$ is the concentration expansion coefficients, the coefficient of Brownian moment $D_{B}$ and the thermophoretic diffusion coefficient $D_{T}$.

### Dimensionless variables

To solve the Equations form (1) to (5), we introduce the following dimensionless variables

6 $$ \begin{aligned} & \psi (x,y,t) = \left( {\frac{{\upsilon_{f} b}}{1 - \gamma t}} \right)^{\frac{1}{2}} x\,F(\eta ),\,\,\,\,u = \frac{\partial \psi }{{\partial y}}\,\,\,\,\,\,{\text{and}}\,\,\,{\text{v}} = - \frac{\partial \psi }{{\partial y}}\,\,,\eta = \left( {\frac{b}{{\upsilon_{f} (1 - \gamma t)}}} \right)^{\frac{1}{2}} y,\, \\ & T(x,y,t) = T_{0} - T_{r} (bx^{2} /2\upsilon_{f} )(1 - \gamma t)^{{\frac{ - 3}{2}}} \Theta (\eta ), \\ & C(x,y,t) = C_{0} - C_{r} (bx^{2} /2\upsilon_{f} )(1 - \gamma t)^{{\frac{ - 3}{2}}} \Phi (\eta ),\,\,\,\,\,\beta = \frac{b}{{\upsilon_{f} (1 - \gamma t)^{\frac{1}{2}} }}h(t).\,\, \\ \end{aligned} $$

By executing Eq. (10), Eqs. (1) to (5) transmuted in the dimensionless form as

7 $$ F^{\prime \prime \prime} + FF^{\prime \prime} - (F^{\prime} )^{2} - S\left( {F^{\prime} + \frac{\eta }{2}F^{\prime \prime} } \right) - MF^{\prime} + {\text{Cos}} \theta \left( {Gr\Theta + \left( {Gr} \right)^{2} \Theta^{2} + Gc\Phi + \left( {Gc} \right)^{2} \Phi^{2} } \right) = 0, $$

8 $$ \Theta ^{\prime\prime} + \Pr \left[ {F\Theta ^{\prime} - 2F^{\prime}\Theta - \frac{S}{2}(3\,\Theta + \eta \,\Theta ^{\prime})} \right] + \Pr Ec(F^{\prime\prime})^{2} + Nt\,\left( {\Theta ^{\prime}} \right)^{2} + Nb\,\Theta ^{\prime}\Phi^{\prime} + M\left( {F^{\prime}} \right)^{2} = 0, $$

9 $$ \Phi^{\prime\prime} + Sc\left[ {F\Phi^{\prime} - 2F^{\prime}\Phi - \frac{S}{2}(3\Phi + \eta \Phi^{\prime})} \right] + \frac{Nt}{{Nb}}\Theta^{\prime\prime} = 0. $$

The non-dimensional terms are measured as

10 $$ \begin{aligned} & S = \frac{\gamma }{b},\,\,\,M = \frac{{\sigma^{*} B_{0}^{2} }}{{\rho_{f} b}},\,\,\,\Pr = \frac{{\mu_{f} c_{p} }}{{k_{f} }},\,\,\,Nt = \frac{{\tau D_{T} (T_{w} - T_{h} )}}{{\upsilon T_{h} }},Gr = \frac{{g\beta_{T} (T_{w} - T_{h} )}}{{bu_{w} }},\, \\ & Gc = \frac{{g\beta_{C} (C_{w} - C_{h} )}}{{bu_{w} }},Ec = \frac{{U_{w}^{2} }}{{c_{p} (T_{w} - T_{h} )}},Nb = \frac{{\tau D_{B} (C_{w} - C_{h} )}}{\upsilon },Sc = \frac{\upsilon }{{D_{B} }}.\, \\ \end{aligned} $$

Here, $S,\,\,\,M,\,\,\,\Pr ,\,\,\,Nt,Gr,\,Ec,Nb,Sc,$ stand for the unsteadiness parameter, Magnetic parameter, Prandtl number, Thermophoresis parameter, Grashof number, Eckert number, Brownian motion parameter and Schmidt number respectively.

And the associated non-dimensional boundary conditions are

11 $$ \begin{aligned} & F(0) = 0,\,\,\,\,\,\,\,F(\beta ) = \frac{S\beta }{2},\,\,\,\,\,\,\,\,\,F^{\prime}(0) = 1,\,\,\,\,\,\,\,\,\,\,\,\,F^{\prime\prime}\left( \beta \right) = 0,\,\,\,\, \\ & \Theta (0) = 1,\,\,\,\,\,\,\,\,\,\Theta ^{\prime}(\beta ) = 0,\,\,\,\,\,\,\,\,\,\,\,\,\,\Phi (0) = 1,\,\,\,\,\,\,\,\,\,\,\,\,\,\,\Phi^{\prime}(\beta ) = 0. \\ \end{aligned} $$

### Physical quantities

Interestingly the important physical measurements like $C_{fx}$ (Surface drag), $Nu_{x}$(rate of heat transport) and $Shu_{x}$ (rate of mass transport) define as^3^

12 $$ \begin{aligned} & {\text{Re}}_{x}^{\frac{1}{2}} C_{fx} = F^{\prime\prime}\left( 0 \right),\,\,\,\,\, \\ & {\text{Re}}_{x}^{{\frac{ - 1}{2}}} Nu_{x} = \Theta^{\prime}\left( 0 \right), \\ & {\text{Re}}_{x}^{{\frac{ - 1}{2}}} Shu_{x} = \Phi^{\prime}\left( 0 \right). \\ \end{aligned} $$

## Solution procedure

The resulting differential expressions are strongly non-linear in many physical issues. Researchers and scientists face difficulties in computing analytical or numerical solutions to such problems. The homotopy analysis method (HAM) is among the most effective computational techniques for computing the sequence solution of nonlinear partial and ordinary differential equations. This approach can be used to solve strongly nonlinear phenomena with no need for a smaller or larger variable. This approach gives you a lot of flexibility in terms of choosing and adjusting the convergence area and estimation rate. The homotopy analysis approach has an advantage over traditional computational approaches in that it prevents rounded off mistakes generated by the discretization process. This approach has been widely applied in a number of nonlinear science and engineering problems^39–43^. Let's make some accurate predictions about $f,\,\,\Theta \,\,\,\,{\text{and}}\,\,\,\Phi$ profiles.

13 $$ F_{0} (\eta ) = \frac{3(2 - S)}{{2\beta^{2} }}\left( {\frac{{\eta^{3} }}{6} - \frac{{\beta \eta^{2} }}{2}} \right) + \eta ,\,\,\,\,\Theta_{0} (\eta ) = 1,\,\,\,\,\Phi_{0} (\eta ) = 1. $$

The linear operators $\pi_{f}$, $\pi_{\Theta }$ and $\pi_{\Phi }$ are presented as,

14 $$ \pi_{f} = \frac{{\partial^{4} F}}{{\partial \eta^{4} }},\,\,\,\,\,\pi_{\Theta } = \frac{{\partial^{2} \Theta }}{{\partial \eta^{2} }},\,\,\,\,\pi_{\Phi } = \frac{{\partial^{2} \Phi }}{{\partial \eta^{2} }}. $$

The expand form of $\pi_{f}$, $\pi_{\Theta }$ and $\pi_{\Phi }$ are,

15 $$ \pi_{F} (\chi_{1} + \chi_{2} \eta + \chi_{3} \eta^{2} ) = 0,\,\,\,\pi_{\Theta } (\chi_{4} + \chi_{5} \eta ) = 0\,,\,\pi_{\Phi } (\chi_{6} + \chi_{7} \eta ) = 0, $$

Expand by utilizing the Taylor’s series as:

16 $$ F(\eta ;\,\,\rho ) = F_{0} \,(\eta ) + \,\,\sum\limits_{x = 1}^{\infty } {\rho^{x} \,\,F_{x} \,\,(\eta )\,,} $$

17 $$ \Theta \,(\eta ;\rho ) = \Theta_{0} \,(\eta ) + \sum\limits_{x = 1}^{\infty } {\rho^{x} \,\,\Theta_{x} \,\,(\eta ),} $$

18 $$ \Phi \,\,(\eta ;\rho ) = \Phi_{0} \,(\eta ) + \sum\limits_{x = 1}^{\infty } {\rho^{x} \,\,\Phi_{x} (\eta ),} $$

Now

19 $$ \begin{aligned} & F_{x} (\eta ) = \frac{1}{x}.\frac{d\,f\,(\eta ;\rho )}{{d\eta }}|_{\rho = 0} ,\,\,\,\,\,\,\Theta_{x} (\eta ) = \frac{1}{x}.\frac{d\,\Theta \,(\eta ;\rho )}{{d\eta }}|_{\rho = 0} ,\,\, \\ & \Phi_{x} (\eta ) = \frac{1}{x}.\frac{d\,\Phi \,(\eta ;\rho )}{{d\eta }}|_{\rho = 0} . \\ \end{aligned} $$

The equation scheme can be described as follows:

20 $$ L_{F} \left[ {F_{x} (\eta ) - N_{x} F_{x - 1} (\eta )} \right] = \pi_{F} R_{x}^{F} (\eta ), $$

21 $$ L_{\Theta } \left[ {\Theta_{x} (\eta ) - N_{x} \Theta_{x - 1} (\eta )} \right] = \pi_{\Theta } R_{x}^{\Theta } (\eta ), $$

22 $$ L_{\Phi } \left[ {\Phi_{x} (\eta ) - N_{x} \Phi_{x - 1} (\eta )} \right] = \pi_{\Phi } R_{x}^{\Phi } (\eta ). $$

where $N_{x} = 0$ if $\rho \le 1$ and if $\rho > 1.$

## Results and discussion

**Figure 2:**
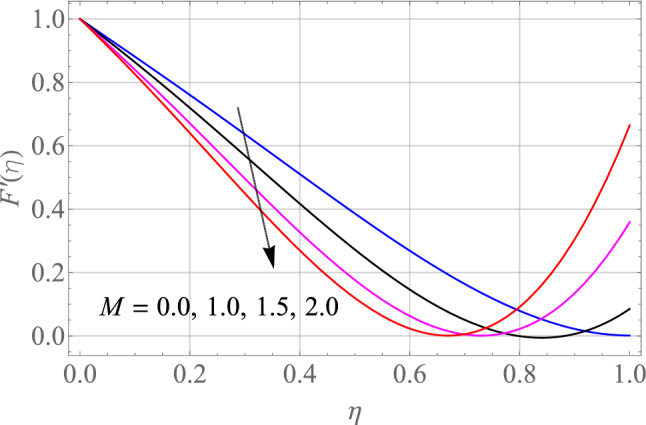
Alterations in $F^{\prime}\left( \eta \right)$ as $M$ raises. When $S = 0.1,\,\Pr = 6.2,\,Gr = Gc = 0.2,Ec = 1,Nb = Nt = 0.3,Sc = 0.3$.

**Figure 3:**
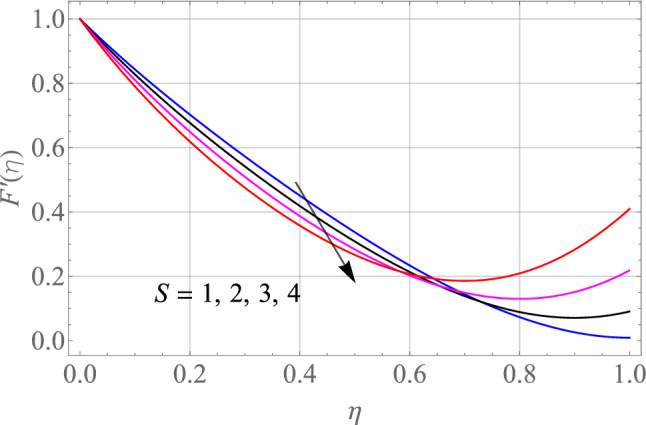
Alterations in $F^{\prime}\left( \eta \right)$ as $S$ raises. When $M = 0.1,\,\Pr = 6.2,\,Gr = Gc = 0.2,Ec = 1,Nb = Nt = 0.3,Sc = 0.3$.

**Figure 4:**
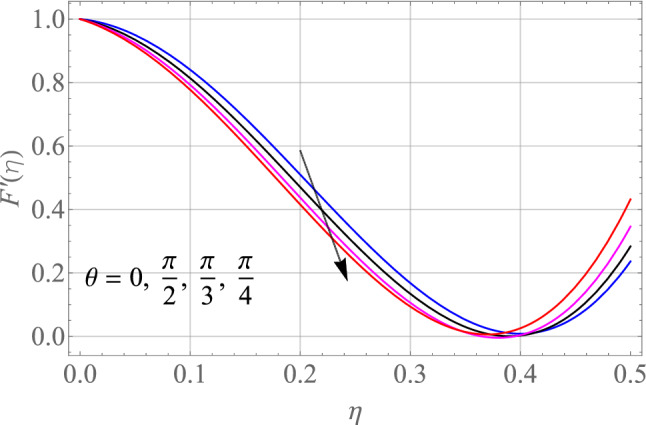
Alterations in $F^{\prime}\left( \eta \right)$ as $\theta$ raises. When $M = S = 0.1,\,\Pr = 6.2,\,Gr = Gc = 0.2,Ec = 1,Nb = Nt = 0.3,Sc = 0.3$.

**Figure 5:**
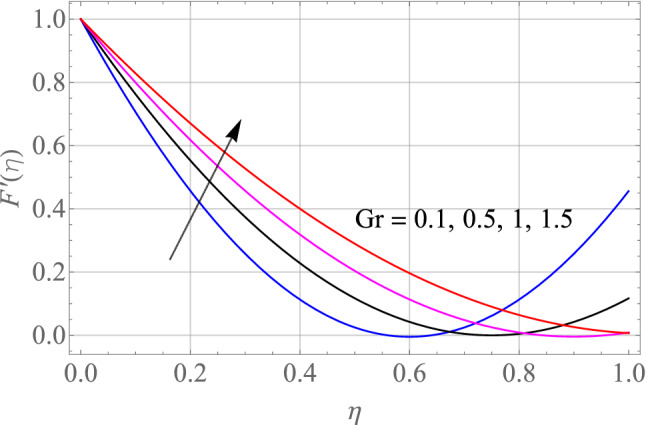
Alterations in $F^{\prime}\left( \eta \right)$ as $Gr$ raises. When $M = S = 0.1,\,\Pr = 6.2,\,Gc = 0.2,Ec = 1,Nb = Nt = 0.3,Sc = 0.3$.

**Figure 6:**
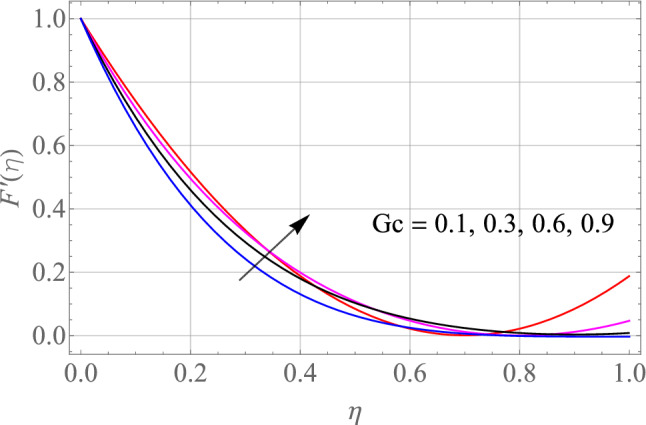
Alterations in $F^{\prime}\left( \eta \right)$ as $Gc$ raises. When $M = S = 0.1,\,\Pr = 6.2,\,Gr = 0.2,Ec = 1,Nb = Nt = 0.3,Sc = 0.3$.

In current portion, we've revealed the physical understanding of a variety of variables that appear in our model such as $$M,\,\,\,S,\,\,\,Nt,\,\,\,Nb,\,\,\,Sc,\,\,\,Ec,\,\,\,Gr\,\,{\text{and}}\,\,\,Gc$$. Although the variables are varied over the adjustment as seen in the detail, we have picked certain design parameters for the sake of our calculating components, such as $$M = 0.3,\,S = 0.2,$$$$\,Nt = 0.3,\,Nb = 0.2,$$ $$\,Sc = 0.1,\,Ec = 1,\,$$$$Gr\, = 0.5\,{\text{and}}\,\,\,Gc = 0.5$$. The effect of different model factors arising in the problem such as $$M,\,S,\,Nt,\,Nb$$ and $$\Omega$$ on $$F^{\prime}\left( \eta \right)$$ is seen in Figs. 2, 3, 4, 5 and 6. Figure 2 depicts the difference in $$F^{\prime}\left( \eta \right)$$ profile with accelerating values of M (magnetic parameter). It is shown that higher the amount of M causes an asymptotic drop in the $$F^{\prime}\left( \eta \right)$$ velocity of a thin liquid film. The basic physics behind such phenomenon is a powerful resistive forec (Lorentz-force) effect and a very slow induction in highly conductive liquid formed by the application of the magnetic field with small attractive Reynolds numbers, leading to improved the frictional influence, velocity drag and consequently flow of thin film liquid deceleration. Figure 3 demonstrates the influence of S (unstability factor) on $$F^{\prime}\left( \eta \right)$$ profile. It is found from the plot that the $$F^{\prime}\left( \eta \right)$$ profile is enlarged slowly by an increment in the magnitude of S which leads to improve the momentum boundary film viscosity. In Fig. 4, the importance of $$\Omega$$ (angle of inclination) is represented on the $$F^{\prime}\left( \eta \right)$$ resulting velocity profile. The rise in $$\Omega$$ is likely to reduce the resulting $$F^{\prime}\left( \eta \right)$$ profiles. We may then assume that there is the opposite relationship between the $$F^{\prime}\left( \eta \right)$$ and the $$\Omega$$. Therefore, the gravity effect decreases for the larger $$\Omega$$ which takes the decreasing velocity field pattern into the boundary layer range.

The special effects of $Gr$(Thermal buoyancy factors) on the resulting $F^{\prime}\left( \eta \right)$ velocity is visualized in Fig. 5. With a growing $Gr$, the velocity of thin film fluid escalates. The ratio of thermal buoyancy forces to viscous forces is known as the $Gr$. As a result, the concentrations of thermal forces grow with increase in the magnitude of thermal $Gr$. The buoyancy forces are produced by huge amounts of Grashof number. It is given in order to improve the produced flow of thin film liquid. As a result, the velocity of fluid increases. Hence, the $Gr$ increases, the thickness of the resultant momentum boundary layer also increases. Figure 6 depicts the unique influence of $Gc$(concentration Buoyancy factor) on the resulting velocity. The velocity of thin film fluid rises as the $Gc$(concentration Buoyancy factor) surges. The ratio of concentration buoyancy forces to viscous forces is known as the $Gc$. As a result, the concentrations of solutal forces grow with increase in the magnitude of concentration $Gc$. The buoyancy forces are produced by huge amounts of Grashof number. It is given in order to improve the produced flow of thin film liquid. As a result, the velocity of fluid increases. Hence, the $Gc$ increases, the thickness of the resultant momentum boundary layer also increases.

**Figure 7:**
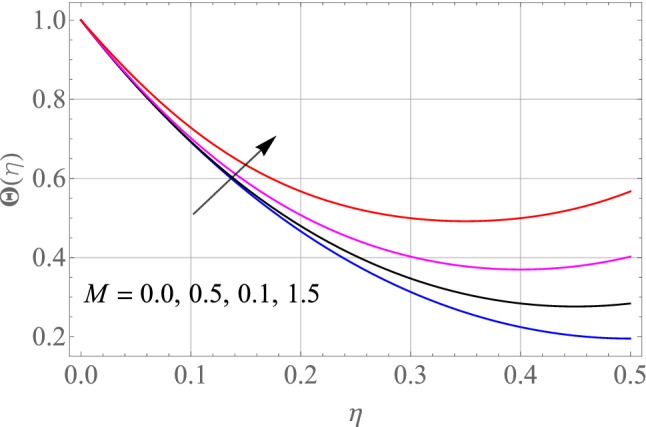
Alterations in $\Theta \left( \eta \right)$ as $M$ raises. When $S = 0.1,\,\Pr = 6.2,\,Gc = Gr = 0.2,Ec = 1,Nb = Nt = 0.3,Sc = 0.3$.

**Figure 8:**
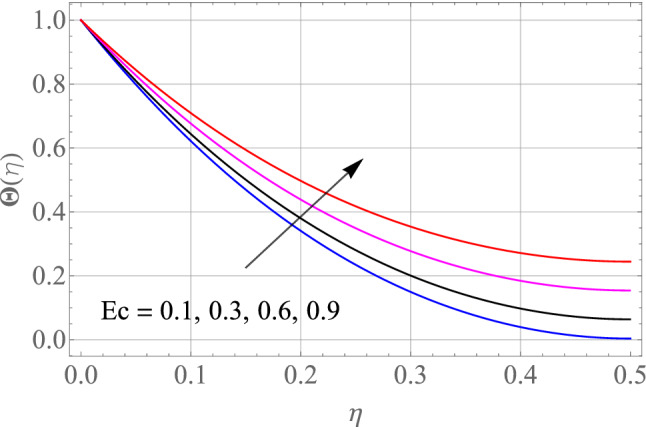
Alterations in $\Theta \left( \eta \right)$ as Ec raises. When $M = S = 0.1,\,\Pr = 6.2,\,Gc = Gr = 0.2,Nb = Nt = 0.3,Sc = 0.3$.

**Figure 9:**
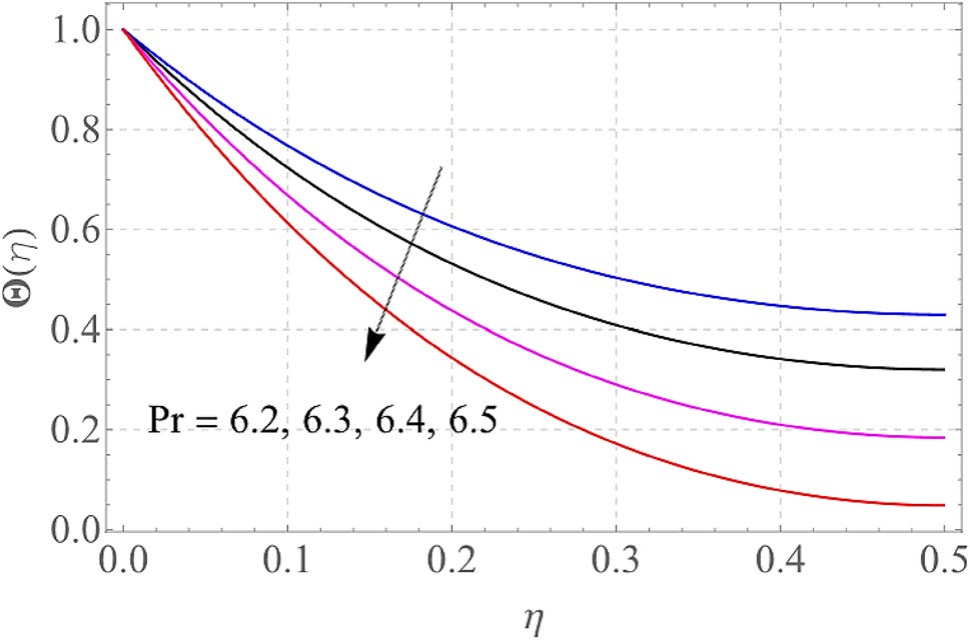
Alterations in $\Theta \left( \eta \right)$ as $\Pr$ raises. When $M = S = 0.1,\,Gc = 0.2,Ec = 1,Nb = Nt = 0.3,Sc = 0.3$.

**Figure 10:**
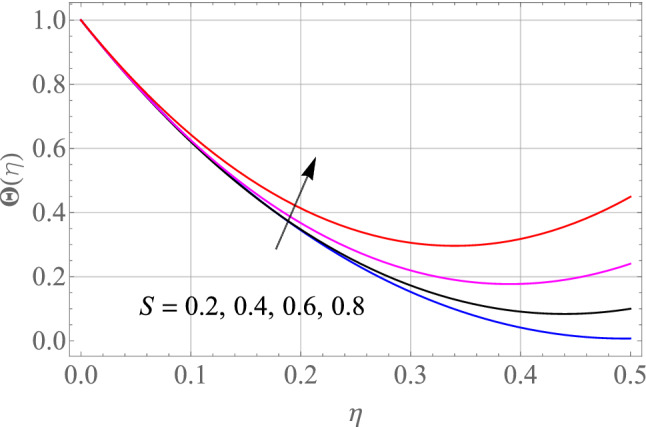
Alterations in $\Theta \left( \eta \right)$ as S raises. When $M = 0.1,\,\Pr = 6.2,Gc = 0.2,Ec = 1,Nb = Nt = 0.3,Sc = 0.3$.

**Figure 11:**
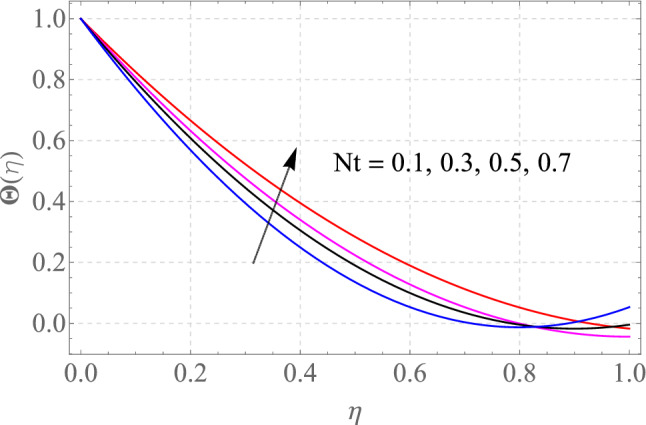
Alterations in $\Theta \left( \eta \right)$ as $Nt$ raises. When $M = S = 0.1,\,\Pr = 6.2,Gc = 0.2,Ec = 1,Nb = 0.3,Sc = 0.3$.

**Figure 12:**
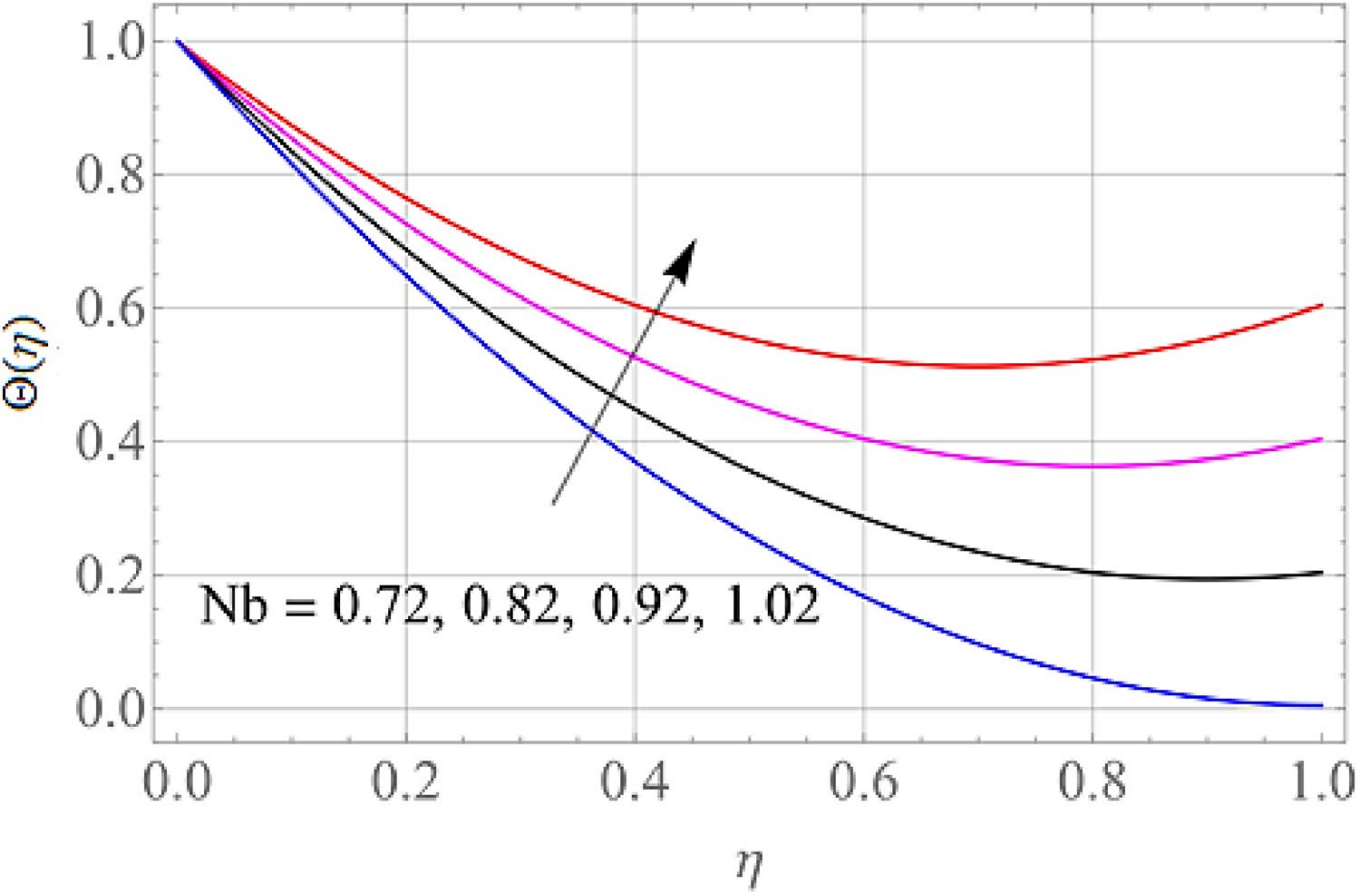
Alterations in $\Theta \left( \eta \right)$ as $Nb,$ raises. When $M = S = 0.1,\,\Pr = 6.2,Gc = 0.2,Ec = 1,Nt = 0.3,Sc = 0.3$.

Figures 7, 8, 9, 10, 11 and 12 demonstrate the temperature profile $\Theta \left( \eta \right)$ for various values of model variables like M, Ec, Pr, S, Nt and N_b_. In Fig. 7, the impact of the M (Magnetic factor) on the resulting $\Theta \left( \eta \right)$(Temperature distribution) is plotted. From Fig. 7 we observed that liquid film temperature rises as the magnetic parameter's strenght is increased. Physically, the Lorentz force, a resistive sort of powers, opposes liquid film movement, causing heat to be produced and, as a result, temperature of liquid and the resulting boundary-layer thickness to thicken. The effect of different values of Ec on thin film nanofluid $\Theta \left( \eta \right)$ temperature profiles is depicted in Fig. 8. It's easy to see how growing Ec magnitude, improves the $\Theta \left( \eta \right)$ in the boundary layer. As Ec is increased, a bump appears near the layer, and the nanofluid temperature then moves to the atmospheric temperature value far from the surface. Basically, the kinetic energy to enthalpy ratio is called the Ec. Figure 9 depicts the effect of Pr on the $\Theta \left( \eta \right)$(Temperature distribution) of liquid films. The opposite finding on the $\Theta \left( \eta \right)$ against the Pr can be seen in Fig. 9. The ratio of momentum to thermal diffusivity is denoted by Pr. The raise in Prandtl numbers indicates that the higher dynamic diffusiveness drops the temperature of fluid. This reality clearly shows that fluid temperatures are increasing strictly in the area of the frontier layer under the increased influence of thermal diffusivity. The importance of the $\Theta \left( \eta \right)$(Temperature distribution) on the S (unsteadiness parameter) is predicted in Fig. 10. It's worth noting that the liquid film temperature is unaffected by the S. For increasing the values of the unsteadiness parameter A in the boundary layer, a small temperature increase is illustrated. Figure 11 determines the influence of N_t_ (Thermophoresis factor) on liquid film $\Theta \left( \eta \right)$ distribution. With defined values of N_t_, an increasing pattern in temperature can be seen. The thermophoresis hypothesis is essentially dependent on the movement of accelerating objects to a cooler area. The movement of fluid objects from a hot environment causes temperature fluctuations. Similarly, Fig. 12 validates the influence of N_b_ (Brownian factor) on liquid film temperature $\Theta \left( \eta \right)$ profile. $\Theta \left( \eta \right)$ rises due to raise in N_b_ factor. Brownian motion is the spontaneous molecular displacement of liquid film that causes the $\Theta \left( \eta \right)$ to reach its peak.

**Figure 13:**
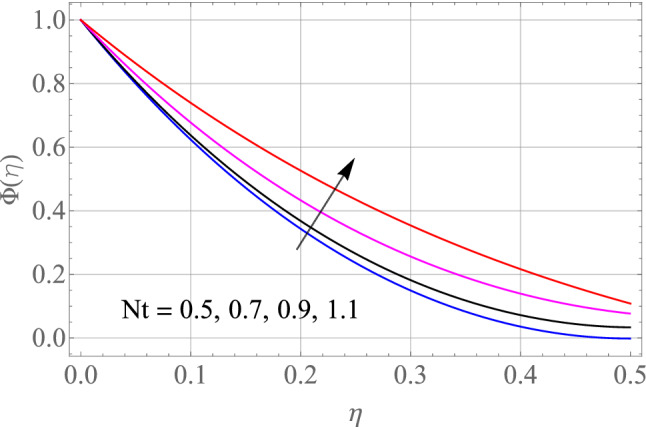
Alterations in $\Phi \left( \eta \right)$ as $Nt$ raises. When $M = S = 0.1,\,\Pr = 6.2,Gc = 0.2,Ec = 1,Nb = 0.3,Sc = 0.3$.

**Figure 14:**
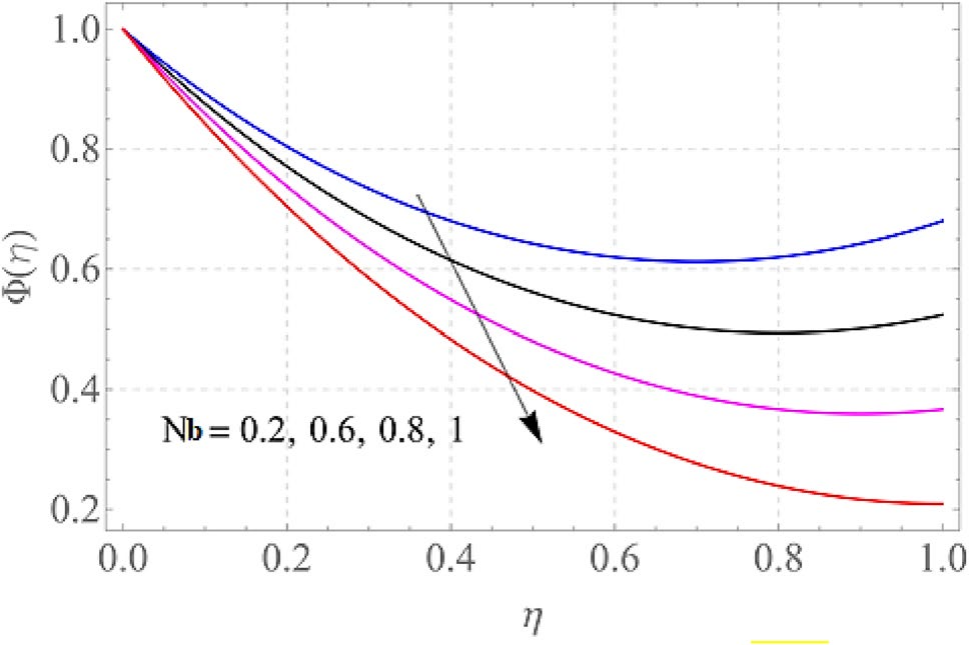
Alterations in $\Phi \left( \eta \right)$ as $Nb$ raises. When $M = S = 0.1,\,\Pr = 6.2,Gc = 0.2,Ec = 1,Nt = 0.3,Sc = 0.3$.

**Figure 15:**
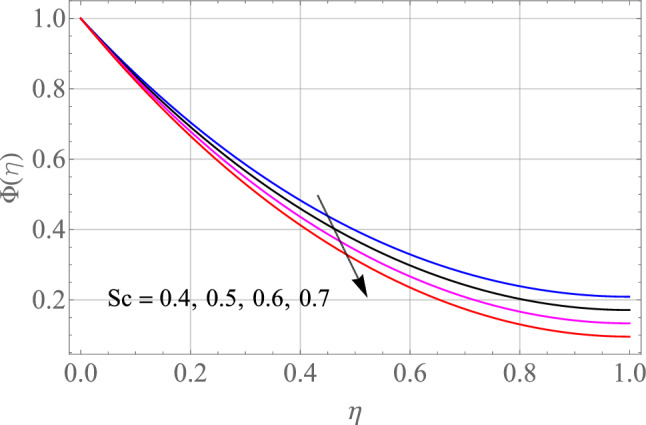
Alterations in $\Phi \left( \eta \right)$ as $Sc$ raises. When $M = S = 0.1,\,\Pr = 6.2,Gc = Gr = 0.2,Ec = 1,Nb = 0.3$.

**Figure 16:**
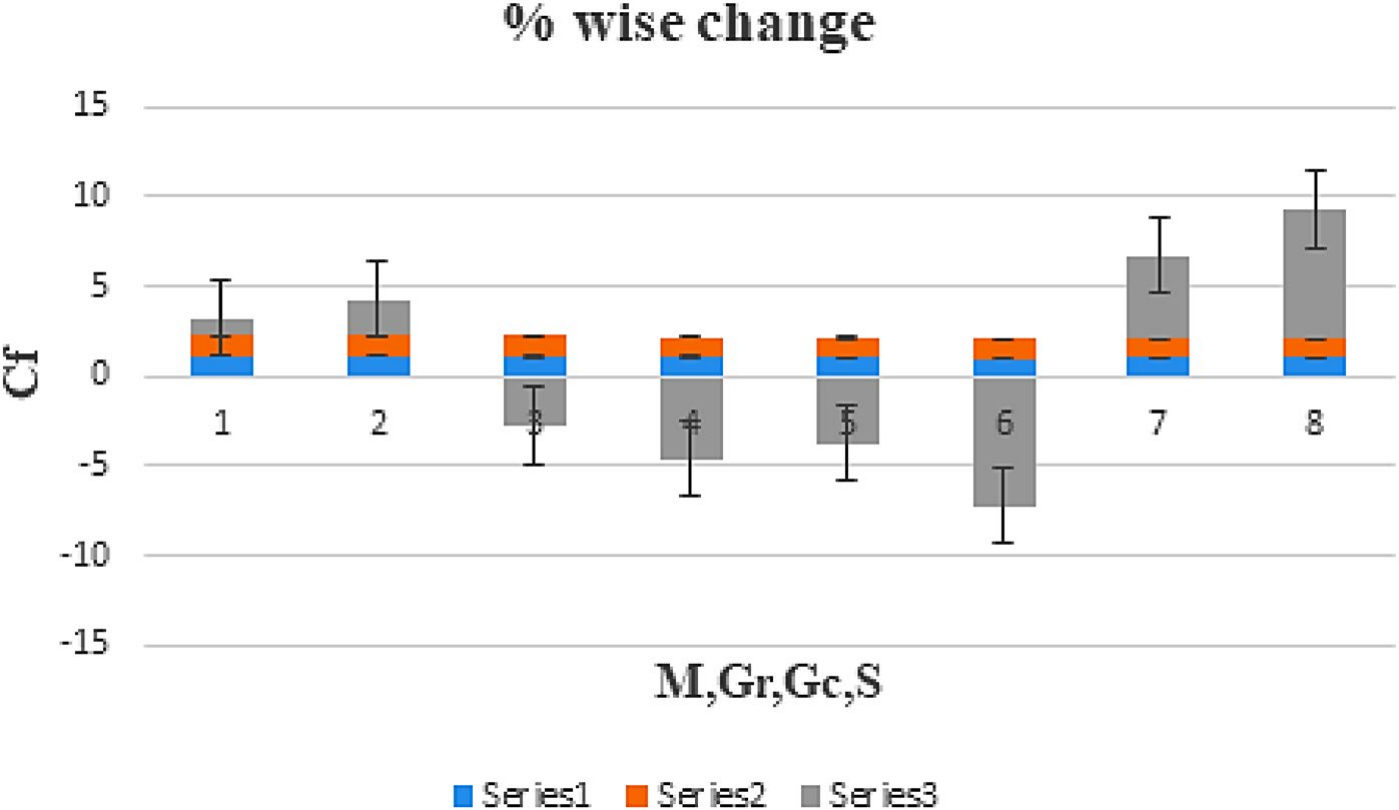
% Wise change in the skin friction coefficient due to the variation In parameters $M,Gr,Gc$. When $\Pr = 6.2,Ec = 1,Nt = Nb = Sc = 0.3$.

**Figure 17:**
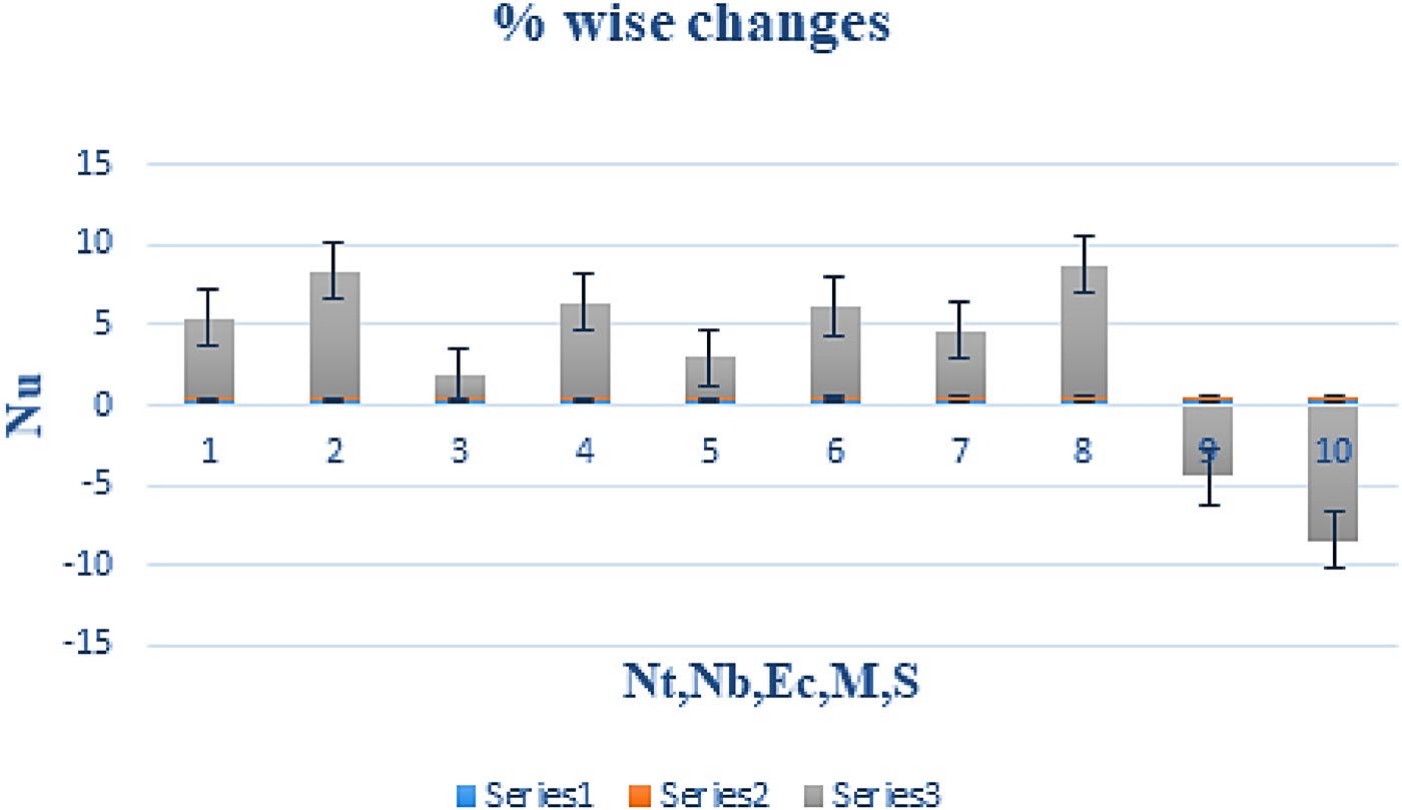
% Wise change in the heat transfer rate due to the variation In parameters $Nt,Nb,Ec,M$ and $S$. When $\Pr = 6.2,Gc = Gr = 0.2,Ec = 1$.

**Figure 18:**
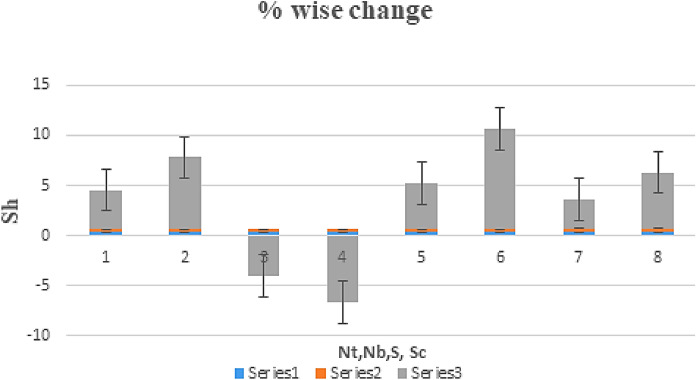
% Wise change in the mass transfer rate due to the variation In parameters $Nt,Nb,S$ and $Sc$. When $M = 0.3,\Pr = 6.2$.

The effect of certain defined parameters like N_t_, N_b_ and Sc on thin liquid film nanofluid concentration profiles in the boundary layer is seen in Figs. 13, 14 and 15. Figure 13 shows the $\Phi \left( \eta \right)$ (Concentration profile) of nanoparticles for various magnitude of N_t_. It has been determined that in the case of concentration distribution within the boundary layer area, N_t_ functions as a supporting variable. This observation arises due to the increase in thermophoretic phenomenon from a physical standpoint. Furthermore, Fig. 14 depicts the $\Phi \left( \eta \right)$ profile for different N_b_ values. The improvement in factor N_b_ corresponds to the occurrence of regular collisions among nanomaterials. As a result, the interaction within nanomaterials drops, resulting in a smaller concentration range. The greater Schmidt number Sc is responsible for decreasing the concentration inside the control volume, resulting in thinning the density of the $\Phi \left( \eta \right)$ (nanoparticle concentration) boundary layer, as shown in Fig. 15. The greater the Schimdt number, the lower the mass diffusivity, and the lower the $\Phi \left( \eta \right)$ in the boundary layer. The % wise variations in the important physical parameters of skin friction, Nusselt number and Sherwood number has been displayed in Figs. 16, 17 and 18 respectively. The % wise variation in these figures are based on the calculated data as mentioned in Tables 1, 2 and 3. The influence of $M,\,\,Gr,Gc,S$ on $C_{f}$ (drag force coefficients) is intended in Table 1. $M$ and $S$ are seen to progress the drag force, however, the reverse consequence is seen for $Gr$ and $Gc$. Table 2 is displayed to attain the influence on the Nusselt number $Nu$ for operating parameters like $Nt,Nb,Ec,M,\,S$. The larger magnitude of $Nt,Nb,Ec,$ and $M$ increase the $Nu$, but, $S$ declines it. Tables 3 is organized to attain the influence of $Nt,Nb,Sc,\,S$ on the mass transfer rate. The increasing values of $Nt,Sc,\,S$ enhancing the mass transfer rate while the decline effect received for the larger values of $Nb$. The present results are validated with the published work and shown in Table 4.

**Table 1:** $C_{fx} Re_{x}^{0.5}$ numerical values versus various parameters.

$M$	$Gr$	$Gc$	$S$	$Cf_{x}$
0.1	0.1	0.1	0.1	1.1121342106
0.2				1.12334321324
0.3				1.13453214576
	0.2			1.1032451762
	0.3			1.0823542313
		0.2		1.0421143211
		0.3		1.0043253214
			0.2	1.0514243212
			0.3	1.0716344213

**Table 2:** $Nu_{x} Re_{x}^{ - 0.5}$ numerical values versus various parameters.

$Nt$	$Nb$	$Ec$	$M$	$S$	$Nu_{x}$
0.1	0.1	0.1	0.1	1	0.20135321234
0.2					0.211464323446
0.3					0.21736321453
	0.2				0.2202331241
	0.3				0.2302133214321
		2			0.2360321754321
		4			0.2432543211
			0.2		0.2532132143
			0.3		0.2633242121
				2	0.251734543213
				3	0.24112342161

**Table 3:** $Shu_{x} Re_{x}^{ - 0.5}$ numerical values versus various parameters.

$Nt$	$Nb$	$S$	$Sc$	$Shu_{x}$
0.1	0.1	0.1	0.1	0.31213416421
0.2				0.324322464323
0.3				0.334423363214
	0.2			0.321012331241
	0.3			0.3121421332143
		2		0.32643217543
		4		0.343239432543
			0.2	0.35395321321
			0.3	0.3626332421

**Table 4:** Comparison of the current results and published work considering common parameters.

$S$	$\beta$	$C_{fx} Re_{x}^{0.5}$ Wang^1^	$C_{fx} Re_{x}^{0.5}$ Qasim^3^	$C_{fx} Re_{x}^{0.5}$ Present	$Nu_{x} Re_{x}^{ - 0.5}$ Wang^1^	$Nu_{x} Re_{x}^{ - 0.5}$ Qasim^3^	$Nu_{x} Re_{x}^{ - 0.5}$ Present
0.1	1.01	1.1121354	1.1121361	1.1121342	0.1530174	0.1530181	0.1530162
0.2	0.87	1.0514256	1.0514263	1.0514243	0.14258763	0.14258764	0.14258751
0.3	0.75	1.0716355	1.0716357	1.0716344	0.1324252	0.1324265	0.1324241

Furthermore, $(\beta = 1.01,0.87,0.75,0.68)$ varies only with velocity profile to sustain the convergence.

## Conclusion

The key goal of this study is to investigate the influences and features of magnetohydrodynamic thermophoresis and Brownian motion of unsteady 2D non-linear convective flow of thin film nanofluid over an inclined stretching sheet. The dynamic model of equations, which are consisting nonlinear coupled PDEs, are translated into ODEs with the aid of resemblance conversion quantities. The HAM procedure is used to solve the corresponding ODEs analytically. A few plots and tables depict the visual effects of different flow factors. Following are the major facts of the current investigation:

- M causes thin film nanofluid motion to be delayed. On the other hand, M has a propensity to raise the $\Theta \left( \eta \right)$ profile.
- $F^{\prime}\left( \eta \right)$ is a decreasing function of S (unsteadiness factor), but S has no effect on $\Theta \left( \eta \right)$.
- The thin film motion is improved when the values of $Gr$ and $Gc$ rises.
- Nb causes the system concentration to drop, while Nt causes the system concentration to grow.
- The % wise increase and decrease in the skin friction, heat transfer rate and mass transfer rate have been observed for each parameter.

## Data Availability

The data that support the findings of this study are available from the corresponding author upon reasonable request.
